# Assessing Environmental Sustainability in Acute Care Hospitals: A Survey-Based Snapshot from an Italian Regional Health System

**DOI:** 10.3390/ijerph23010020

**Published:** 2025-12-22

**Authors:** Andrea Brambilla, Roberta Poli, Michele Dolcini, Beatrice Pattaro, Stefano Capolongo

**Affiliations:** 1 Design & Health Lab, ABC Department, Politecnico di Milano, 20156 Milano, Italy; andrea1.brambilla@polimi.it (A.B.); beatrice.pattaro@polimi.it (B.P.); stefano.capolongo@polimi.it (S.C.); 2 School of Architecture and Urban Design, Politecnico di Milano, 20156 Milano, Italy; roberta1.poli@mail.polimi.it

**Keywords:** environmental sustainability, hospital facilities, hospital design, next-generation hospital, hospital planning

## Abstract

Background: The healthcare sector plays a significant role in environmental degradation, particularly through energy consumption, emissions, and resource use associated with hospital operations. Despite growing global awareness of the impacts, environmental sustainability remains only partially embedded with the design, planning, management, and evaluation of hospital facilities, and empirical evidence is still limited. Methods: This exploratory study employed a mixed-method, two-phase approach. First, a scoping literature review identified key environmental dimensions and approaches for environmental sustainability in hospitals infrastructures. Second, a structured survey was distributed to Italian hospitals from Lombardy Region, between May and June 2024, to assess environmental performance and environmental strategy adoption. Results: Eight (n = 8) core environmental sustainability dimensions emerged from the review: energy efficiency, resource and waste management, transportation and mobility, materials and construction, environmental compliance, emissions, site sustainability, and design strategies. The subsequent based on these dimensions, gathered responses from (n = 18) healthcare facilities from Lombardy region, Italy. Findings revealed substantial gaps, since key measures such as on-site renewable capacity, water reuse systems, environmental certification application and health-island mitigation practices appear to be adopted sporadically. In addition, many of the surveyed facilities show consumption levels that exceed the benchmarks outlined in the literature. Discussion: The findings of this study reveal a notable misalignment between the sustainability debate, maturity promoted in the academic literature and the actual practices implemented in the Italian regional context. This mismatch highlights the importance of developing more uniform evaluation tools, policy requirements, and strengthening the organizational capabilities, to improve environmental performance in Italian hospital facilities.

## 1. Introduction

The increasing environmental burden of healthcare has led to growing advocacy for embedding sustainability as a core dimension of health system performance. Previous studies have advocated the measurement of environmental impacts as a quality dimension of healthcare, alongside patient care, equity, cost, and workforce wellbeing [1,2]. Healthcare infrastructures represent a fundamental cornerstone for maintaining and promoting the health of the population. There is a growing recognition that healthcare capital stock and physical infrastructure constitute a fundamental component of healthcare quality [3]. At the same time, however, facilities such as hospitals are among those that consume the most energy and have the greatest environmental impact [4]; this is due to several factors, including the fact that their operation must be guaranteed around the clock, without being able to afford to interrupt operations even in emergency situations. In addition, hospital complexes deal with frail users for whom specific and differentiated thermo-hygrometric conditions must be guaranteed according to the different functional units within the facility itself [5,6]. Hospitals are often and increasingly characterized by large dimensions on which depend a directly proportional consumption of resources, and consequently a proportional environmental impact. Carbon dioxide (CO_2_) emissions in the health sector account for 5.2% of global emissions, as reported in the 2022 Report of the Lancet Countdown on Health and Climate Change [7]. The negative impact is not limited only to air pollution; in fact, water and soil polluting factors are also present, as well as the production of solid and liquid waste and the use of valuable resources such as drinking water or non-renewable resources [8]. All this has a negative impact not only on the environment but also on humans; the latter, in fact, is to be considered as an integral part of the environmental ecosystem [9]. Thus, the environmental impacts of hospital facilities contribute to more complex risk factors, such as global warming and climate change [7]. Health facilities are the first and last line of defence against the impacts of climate change, as despite the fact that these produce adverse environmental effects, they provide needed services and care to people harmed by extreme weather and other long-term climate hazards. This contradiction has led to the urgent need to reimagine the design and management models of hospital infrastructure toward environmental sustainability criteria, with the aim of mitigating environmental impacts and ensuring greater resilience to climate change [10]. Over 70% of hospital buildings have outlived their life cycle, and the pandemic highlighted the need to design more modern and resilient hospitals, with welcoming and reassuring spaces characterized by flexible and sustainable architecture [11]. Consequently, it is essential to focus on the sustainable development of healthcare infrastructure; this becomes the essential prerequisite for ensuring health, touching on the social, economic, and, in particular, the ecological–environmental aspects of sustainability itself [12,13].

In 2020, the World Health Organization drafted guidance titled “WHO guidance for climate-resilient and environmentally sustainable health care facilities”, in which environmentally sustainable healthcare facilities are defined as those “facilities that improve, maintain or restore health by minimizing negative impacts on the environment and taking advantage of opportunities to restore and improve it.” [14].

Healthcare facilities that are resilient to climate change and ecologically sustainable thus contribute to the improvement of the quality of healthcare and services provided, positively impacting the reduction in costs of the facilities themselves. It can be argued that the pursuit of environmentally sustainable development goals in healthcare facilities, but not only there, brings benefits regarding social and economic sustainability as well [8,15].

The healthcare sector is a fast-changing industry, with trends that are moving rapidly; however, sustainability does not yet seem to be fully part of these trends in the hospital setting. The definition of sustainable hospital infrastructure is still hampered by the absence of unambiguous guidelines and the presence of inconsistent and outdated regulations. Although to date there are tools for assessing these elements, such as ISO 14001, EMAS, LEED, and BREEAM, these all appear to be voluntary, and their application is not systematic and universally agreed upon [16]. In this context, it becomes essential to define measurable environmental criteria and shared design tools to guide the ecological transition of the hospital system. Indeed, the purpose of this study is to understand which issues related to environmental sustainability in hospital facilities are most frequently mentioned in the most recent scientific literature and which strategies are explicitly suggested to improve their sustainability.

### 1.1. Aim of the Study and Research Questions

This study investigates environmental sustainability in a real hospital setting, as defined in the recent literature. Based on the results of a scoping literature review, the study develops a framework of sustainability criteria to be adopted to assess the implementation across a sample of acute care hospitals in the region. The study is based on two main research questions:

- Which environmental sustainability dimensions and strategies are most consistently discussed in the contemporary scientific debate on hospital infrastructures?
- What is the state of the art of a sample of Lombardy hospital facilities with respect to the environmental sustainability strategies identified within the literature review?

### 1.2. Research Context

This research focused on the Italian hospital system, and more specifically of the Lombardy region, selected as the primary and representative case study for Italian healthcare. The region was deemed appropriate for an in-depth investigation of the state of the art in hospital sustainability maturity for the following reasons:

- The region presents homogenous climatic conditions and seasonal patterns, allowing for data normalization;
- It is the most populous region in Italy, accounting for approximately 17% of the national population in 2024 [17];
- It includes a wide range of hospital typologies, encompassing both public and private facilities, offering diversity in organizational and infrastructural characteristics [18].

For these reasons, Lombardy constitutes a robust and widely recognized case for empirical analysis, as also acknowledged in previous studies [19,20]. While healthcare systems in Italy are regionally governed and exhibit organizational differences, the scale, diversity, and complexity of Lombardy’s system provide valuable insights that may be indicative of broader national trends.

## 2. Materials and Methods

**Figure 1:**
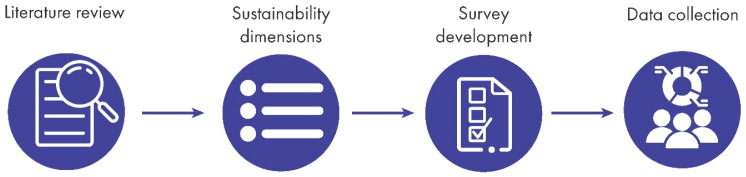
Flowchart of the methodology.

The methodological process followed in the development of this study consisted of two phases. The first consists of a scoping literature review to identify the environmental sustainability dimensions most frequently addressed in recent research on hospital facilities, encompassing both design-related and operational strategies. The second phase involved an empirical study, structured around the criteria deriving from the scoping review and administered to a sample of regional hospitals for empirical analysis (Figure 1).

### 2.1. Literature Review

The scoping scientific literature review was developed with the aim of mapping the main environmental sustainability dimensions relevant to hospital facilities, as well as the strategies proposed to reduce the environmental impacts. The aim was not to systematically synthesise all the evidence, but rather to explore the most recurrent and measurable dimensions in recent scientific debate to build a questionnaire aimed at collecting empirical evidence and primary data from an Italian hospital. The review process considered articles recognized by the international scientific and technical community in the field of environmental sustainability and hospital design. The sources used in this context included the following:

- Scientific literature from relevant databases such as Scopus, Web of Science, and PubMed;
- Grey literature.

In order to identify scientific articles useful for the purpose of this paper, keyword characterized by specific and correct terminology for research purposes are outlined, divided into three levels of definition:

- The first level of keyword definition concerns the physical context in which the research focuses; the words defined for the research are “Hospital” OR “Hospital facilities” OR “Hospital building” OR “Hospital environment”.
- The second level of keyword definition concerns the main content of the research itself, and the words defined are “Environmental sustainability” OR “Ecological sustainability” OR “green sustainability”.
- The third level was used to narrow the content toward a more physical and architectural interpretation of the research; the words are “Design” OR “architecture” OR “built environment”.

The search string was developed based on previously analyzed documentation. The following eligibility criteria were also defined to define the search scope in more detail:

- Papers published after 2019;
- Editorial articles, research papers, or reviews only;
- Areas of research closely related to the environmental sustainability of hospital facilities in architectural settings.

The year 2019 was chosen as a landmark year because it marked important global developments in sustainability: the signing of the U.N. 2030 Agenda on Sustainable Development Goals (SDGs), the International Labor Organization’s report on promoting a greener economy, and the emergence of the European Green Deal. In addition, the COVID-19 pandemic has increased attention on the health sector, bringing hospitals to the centre of public debate.

**Figure 2:**
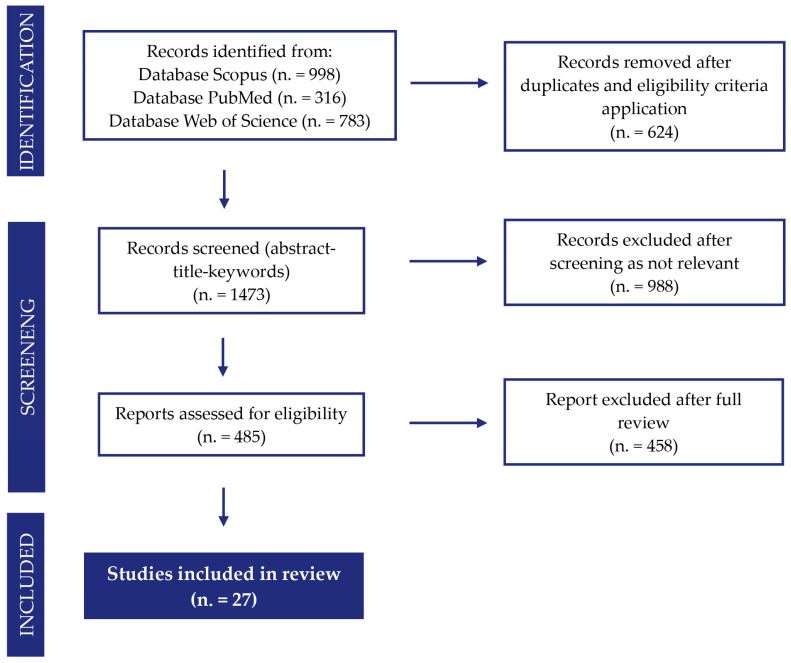
PRISMA chart—literature review.

Using the Scopus database, (n = 998) results were obtained, after removing scientific articles published before the year 2019, (n = 242) duplicates were excluded, and eligibility criteria were applied to remove irrelevant scientific fields (e.g., chemistry, mathematics, computer science, and nursing), obtaining (n = 436) contributions. These articles were analyzed by screening the title, abstract, and keywords; exclusion occurred because the topics covered by some articles and reviews were not relevant to the research field of this paper. This resulted in the exclusion of 414 records deemed irrelevant. At the end of the process, 22 papers were selected (Figure 2).

In order to verify and supplement the previous search, a further investigation was carried out using the same procedure on the PubMed and Web of Science databases, in which 316 and 783 results were obtained, respectively. After applying the screening and eligibility criteria, 2 more papers were selected from the PubMed search and 3 more papers from the Web of Science search, to be added to the 22 previously defined, for a total of 27 papers.

After identifying the 27 papers, all the environmental sustainability-related variables were extracted. These elements were codified by adopting an inductive process, where two authors assigned thematic labels to each criterion emerging from the review of the 27 studies. Through comparison and refinement, overlapping or closely related items were grouped into broader thematic categories. Variables that appeared only sporadically in the literature have been excluded, in line with the scope of an exploratory investigation.

### 2.2. Survey

After identifying useful strategies for achieving environmental sustainability in the hospital setting through the review of scientific literature, a questionnaire consisting of 32 questions was developed. The questions used in the questionnaire can be found in Appendix B. The survey addresses eight (n = 8) criteria related to the environmental sustainability of facilities, as well as an initial part of the questionnaire related to facility biographical information, consisting of 5 questions (Table 1).

The survey was completed by hospital facility managers and officers from the technical department, reflecting their primary involvement in environmental performance management in healthcare organizations. The responses to the questionnaire revealed different types of facilities built between 1837 and 2010. The facilities that participated in the data collection are characterized by three different levels of complexity, in accordance with Ministerial Decree No. 70 of 2 April 2015: eleven facilities have a basic level, four facilities have a first emergency level (DEA I), and three hospitals have a second emergency level (DEA II). Questionnaire responses were structured according to the topic being investigated, but most involved a binary (yes/no) or multiple-choice response. There was a minority of questions for which a short open-ended response was provided.

The questionnaire was sent to 135 hospital health facilities located within the Italian region of Lombardy. All other types of healthcare facilities were excluded. The questionnaire was administered as a form that could be filled out online, as well as in tabular spreadsheet or PDF format that could be printed as needed.

- Online: The questionnaire was sent via a link attached to an introductory e-mail.
- Table spreadsheet/ PDF format: Upon request, the questionnaire was provided in tabular spreadsheet or PDF format.

The questionnaire was made available from 13 May 2024 to 30 June 2024. The data from the responses to the questionnaire were analyzed and processed anonymously and confidentially, allowing key characteristics and trends to be extrapolated.

## 3. Results

### 3.1. Literature Review Results

The literature review aimed to identify the main environmental sustainability dimensions for healthcare facilities, with the objective of outlining the most discussed performance areas in recent research, to structure the survey for the empirical investigation. Therefore, the focus was on those articles that proposed operational and measurable solutions, to the exclusion of generic or purely theoretical contributions lacking direct applicability. The scoping analysis of the literature identified eight criteria that were useful for defining environmental sustainability in a hospital setting (Table 2).

- Energy efficiency: Reducing Energy Use Intensity (EUI) and increasing energy production from renewable sources was central. In particular, the importance of integrating photovoltaic, geothermal, and solar thermal systems in hospital buildings was highlighted.
- Resource consumption and waste management: The articles analyzed insist on separate collection, efficient management of hazardous medical waste, and reuse of stormwater and wastewater after proper purification.
- Transport and mobility: The findings highlighted the need to implement policies that promote sustainable mobility solutions, such as bicycle parking, car sharing, and electric vehicle charging stations, while discouraging the reliance on traditional private vehicles for both staff and users.
- Materials and constructions: The choice of environmentally friendly materials that are recycled or recyclable and free of harmful emissions has been identified as one of the key aspects of reducing the overall environmental impact of healthcare buildings.
- Environmental compliance: Voluntary adherence to internationally recognized standards and certifications (LEED, BREEAM, ISO 14001) is cited as critical to guide the sustainable design process.
- GHG emissions: Several contributions stress the importance of emissions reduction, especially CO_2_ and anesthetic gases.
- Site sustainability refers to the hospital’s ability to integrate with the environmental, landscape, and urban context in which it is located. This includes solutions such as the presence of green, open, and draining spaces that not only improve environmental comfort and the local microclimate but also establish a dialogue with the built environment, strengthening the connections between architecture and landscape.
- Design strategies: This is a transversal criterion concerns the application of design strategies aimed at reducing passive energy needs. Importance is given to the orientation of the building, optimized to maximize winter solar gains and reduce summer overheating. Solar shading, ventilated facades, green roofs, and natural ventilation systems are cited as effective tools to improve internal environmental quality, increase thermo-hygrometric comfort, and reduce the energy load of the systems. Furthermore, the importance of the integration between architecture and landscape is underlined, with examples being the design of green courtyards, solar greenhouses, and vegetal buffers, which contribute both to the local microclimate and to the psycho-physical wellbeing of users.

Despite the diversity of the documents examined, a strong convergence emerges regarding the identified environmental sustainability criteria. There is broad consensus on the need to develop the principle that hospital design and management should be grounded in a holistic approach, simultaneously integrating environmental, social, and economic dimensions.

### 3.2. Survey and Sample Description

This section presents the results of the survey, which aimed not only to map the current state of the art of the healthcare situation in Lombardy but also sought to analyze the design, management, and retrofit strategies to be implemented in order to achieve an improvement in environmental sustainability from the perspective of the hospitals of the future [42]. A total of 135 hospitals were contacted, of which 18 responded and participated in the data collection process (Appendix A), yielding a response rate of approximately 13.3% (18/135). The response rate and the resulting sample size are consistent with the aims of an exploratory study. The hospital selection for the study was based on inclusion criteria to ensure relevance and comparability. Only public hospitals with a minimum capacity of 100 beds were considered, as these facilities typically have a broader operational scope and environmental impact compared to small-scale community hospitals. The final sample included eligible hospitals within the Lombardy region meeting these criteria. In particular, the analysis aimed at collecting data on design and management strategies across a set of key environmental sustainability criteria, including energy efficiency, resource consumption and waste management, sustainable transportation and mobility, materials and construction practices, environmental compliance, emissions reduction, and site integration and sustainability, as well as climate-responsive design strategies.

#### State of the Art of the Sample of Hospitals Based on Environmental Sustainability Criteria

**Figure 3:**
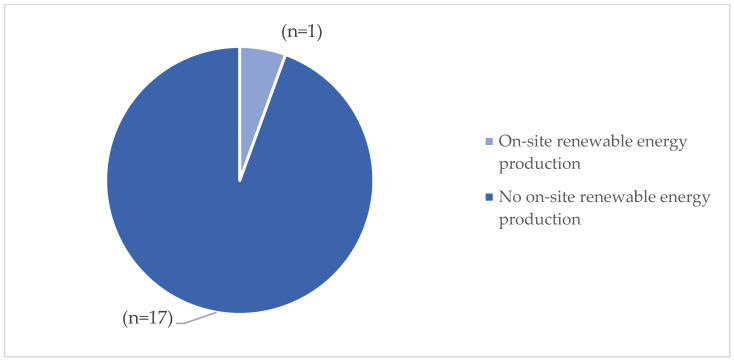
Renewable energy production results.

**Figure 4:**
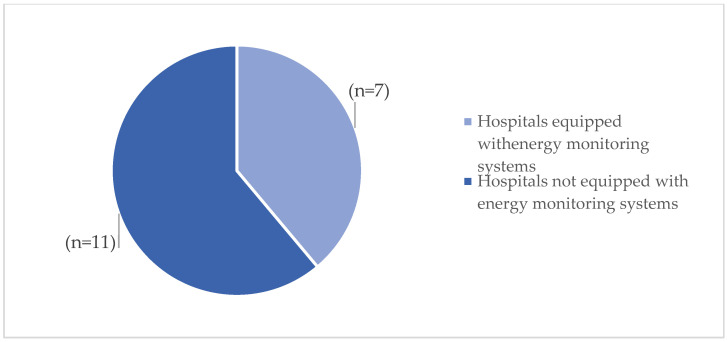
Hospitals equipped with remote energy monitoring systems.

Hospital infrastructures were initially analyzed based on the criterion of energy efficiency. The results show that only (n = 1) of the facilities demonstrated the ability to generate energy from on-site renewable sources (Figure 3). Additionally, only (n = 5) of the facilities have an energy supply contract with a guarantee of origin from renewable sources. Furthermore, 17% (n = 3) of hospitals assessed employ efficient energy management systems, 22% (n = 4) are equipped with remote monitoring for energy consumption, and finally, 40% (n = 7) have energy consumption monitoring systems in place (Figure 4).

Regarding the consideration of the criterion of resource and waste management, an important aspect emerged: the near-total lack of data concerning waste production and recycling. None of the facilities assessed could provide data regarding the share of waste production. Furthermore, no facility was equipped with systems or solutions for the efficient management, purification, or reuse of both rainwater and wastewater.

**Figure 5:**
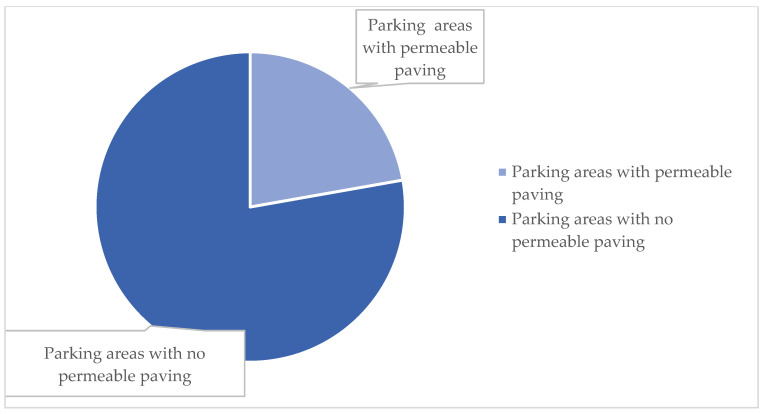
Permeable paving in parking areas.

The criterion related to site sustainability revealed that 72% (n = 13) of hospitals have a percentage of green space relative to the total site area of less than 45%. In eleven cases (61%), however, the percentage of open spaces and total site area exceeds 65%. Only 6% (n = 1) of the facilities have a portion of green roof used for rainwater management, and finally, 22% (n = 4) of the facilities feature a significant portion of parking areas with permeable paving (Figure 5).

Regarding the criterion of transportation and mobility, it emerged that (n = 6) of hospitals are equipped with services for sustainable mobility sharing, (n = 6) of the facilities have at least one parking space for electric vehicle charging, and a number of spaces for light mobility that covers at least 15% of the total workforce attending the facility.

**Figure 6:**
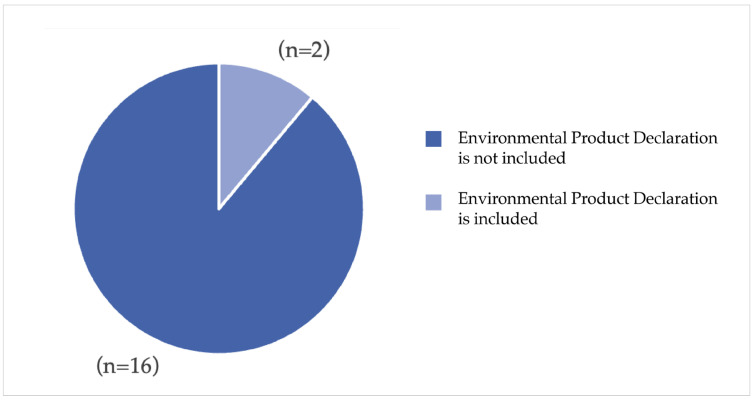
Environmental Product Declaration is included in procurement policies.

Furthermore, 100% (n = 18) of the facilities are characterized by a distance of less than 450 m between the bus stop and the main entrance, while in 33% (n = 6) of the cases, the distance to the nearest public transport station exceeds 700 m from the main entrance. Concerning the sustainability assessment of materials procurement, only (n = 2) of the hospitals included the Environmental Product Declaration (EPD) within their policies (Figure 6).

**Figure 7:**
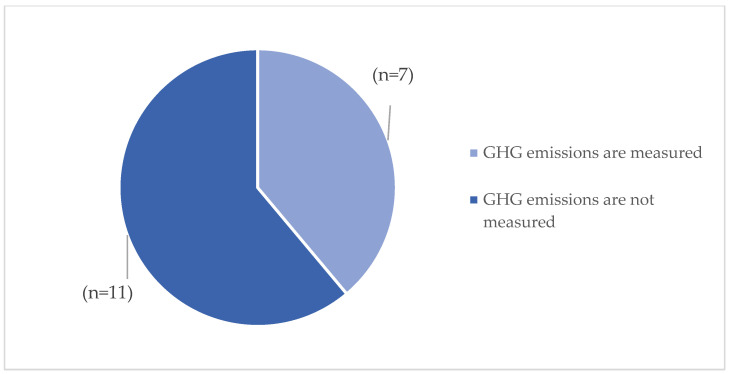
GHG emissions measurement and tracking.

The data collected related to the emissions criterion also yielded limited results, with only 40% (n = 7) of the facilities responding to the question regarding the amount of CO_2_ emissions per square metre per year (kg/sqm/year) and the capability of measuring and tracking emissions over time (Figure 7).

## 4. Discussion

The main objective of the research was to conduct an exhaustive analysis of recent scientific literature in order to identify the most current and recurring themes and strategies for achieving environmental sustainability in the context of hospital infrastructures. Also, the study aimed to assess a sample of Italian hospital facilities to measure the adoption of environmental sustainability strategies and approaches.

An important issue emerged from the analysis of the theoretical background and the literature review: the lack of standardization of the strategies and related threshold values, as well as the evident absence of universally accepted methodologies for the assessment of environmental sustainability. This framework limits comparability between different geographical contexts and different healthcare facilities [11]. Moreover, the survey results highlight an important gap between the best practices proposed in the research and the current state of healthcare infrastructures, revealing critical issues in both the design, management, and resilience of hospital facilities.

**Figure 8:**
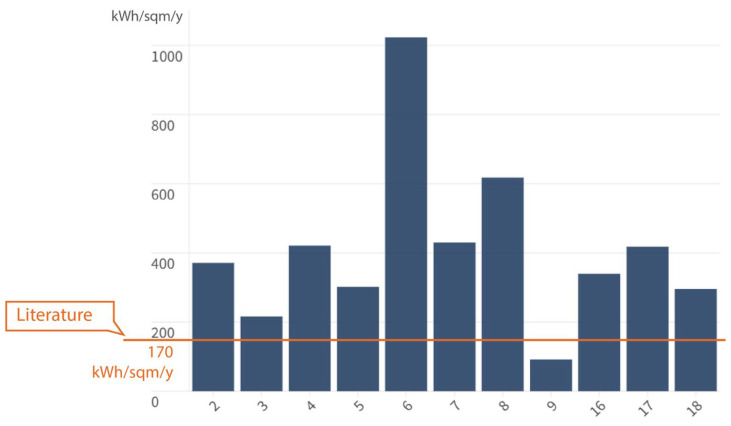
Building Energy Intensity (BEI) values higher than the threshold identified in the literature.

The literature highlights the importance of the energy efficiency criterion in order to achieve environmental sustainability [23,24,25,26,27,28,29,30,31,32,33,34,35,36,39,40,41,43,44,45,46]. However, the survey data indicate a state of the art for the Lombardy sample of cases that is far removed from the good practices defined in scientific theory. Previous studies have highlighted that direct and indirect energy consumption typically ranges between 230 and 330 kWh/m^2^ per year, with consistent values observed across different regions and time periods. While some facilities performed under this higher value (4/18), only (n = 1) performed better than the best practices outlined in the literature for acute hospitals, which is 170 kWh/sqm/year (Figure 8). This case could depend on the fact that it is a compact facility and consequently characterized by a small number of beds (n = 130), as well as a low DEA I level of complexity, and characterized by the presence of only (n = 3) operating theatres. The hospitals showing the highest BEI are generally the largest ones, with an extensive floor area, a greater number of beds (n = 1200), and a DEA II level of complexity, with (n = 36) operating theatres. Other factors that proved to be particularly influential on the BEI are the age of the buildings and the high articulation of the architectural type, which consequently leads to a greater dispersion surface and floor area. BEI was not provided and could not be calculated for case studies 1, 10, 11, 12, 13, 14, and 15.

In general, it can be stated that the absence of solutions for the production and supply of energy from renewable sources, as well as the absence of systems for the management and monitoring of energy consumption, is a major obstacle to achieving environmental sustainability within the Lombardy sample of cases. A second criterion that emerges from the literature review as fundamental to the achievement of environmental sustainability is that of resource consumption and waste management [16,23,24,25,26,27,28,29,34,35,36,38,39,40,43,44,45,46,47,48]. Here again, it emerged from the empirical investigation that most of the hospitals in the sample exceed the threshold value for water consumption of 0.84 mc/sqm/year identified in the theoretical research. Again, it could be seen that hospitals characterized by smaller size, without open spaces, and with a low DEA I complexity level have lower water consumption values than the other cases.

Hospitals with higher water consumption are identified as those with a higher DEA II level of complexity and which consequently have a high number of beds (n = 1116) and a large floor area. The age of the facility and the high level of complexity found in the floor plans also proved influential. In general, it must certainly be emphasized that the absence of efficient management systems has a negative impact on consumption. It should also be emphasized that water consumption depends on certain variables that were not mapped within this research, such as specific medical services that require large amounts of water, an example of which can be mentioned is dialysis.

The third criterion of environmental sustainability that emerged from the literature review is that relating to transport and mobility [21,23,24,25,39,44,45]; in this case, it can be stated that the public transport network in Lombardy is well integrated with the various hospital infrastructures. Less effective are, on the other hand, the solutions relating to light mobility and electric recharging, which are not very present in the hospital buildings.

Regarding the criteria related to materials and construction [23,25,26,27,30,31,34,36,38,39,43,45,48] and environmental compliance [23,24,26,27,28,30,31,32,33,34,35,36,37,38,39,40,43,44,45,46,47], it is not possible to add further considerations given the limited data collected by the survey and the fact that hospitals have not fully implemented these aspects. Another relevant criterion that emerged from the literature is the one referring to emissions [27,31,38,39]. In this case, few data were found; only (n = 6) hospitals were able to answer the question referring to CO_2_ emissions, only one of which exceeded the threshold value defined by the theoretical research and equal to 180 Kg/sqm/year of CO_2_ emissions due to the complexity of the structure and also linked to the energy consumption of the building.

The last criterion to emerge from the literature review is that of site sustainability [26,28,41,43]. Almost all hospitals have a green/total area ratio below the threshold value defined at 45%. There are, however, four exceptions, two of which, although located in large cities, have managed to maintain a good percentage of green areas useful for mitigating heat island effects as well as for the proper management of rainwater, while the other two are located outside urban centres and therefore have large green areas available. The cases with no green areas are located in the very dense urban fabric of provincial capitals and, precisely for this reason, do not have large open spaces.

In the final analysis, it can be pointed out that the ratio of tree-lined/covered/underground parking spaces to total parking spaces fails to reach the threshold value of 80% in any case. This figure is very important in terms of environmental sustainability, as it is an excellent solution for combating the heat island effect, as well as for the management of rainwater, which, by not going directly into the sewer system, can be reused or stored.

Finally, the declared absence of design strategies aimed at resilience to the effects of climate change and the reduction in the negative impacts of anthropization on the environment is an indicator that well summarizes the criticalities and shortcomings of Lombardy’s hospitals, since it encapsulates many of the aspects considered so far [12,25,26,28,30,31,32,33,40,41,43]. This not only highlights the actual presence or absence of solutions aimed at resilience, but above all underlines the fact that hospitals, or those who answered the questionnaire on their behalf, do not feel sufficiently informed or prepared with regard to the issue in question.

## 5. Conclusions

The study aimed to critically assess the implementation of environmental sustainability strategies in hospital infrastructures with a mixed-method approach, comparing the evidence emerging from the scientific literature with real-world practices observed in healthcare facilities across the Lombardy region. The exploratory findings reveal a substantial gap between the sustainability strategies promoted in academic and technical literature and those actually applied in practice in the context analyzed. Key areas such as renewable energy integration, climate resilience, energy monitoring, green procurement, and environmental certification are significantly underdeveloped or inconsistently implemented. While some facilities report ongoing initiatives, these are rarely framed within a comprehensive or standardized sustainability policy. This discrepancy suggests that, despite the growing awareness of environmental issues in the healthcare sector, many hospitals remain limited by structural, regulatory, and cultural barriers that hinder the transition to more sustainable models. The limited diffusion of environmental certifications (e.g., LEED, BREEAM) further reflects the absence of strong institutional mandates or performance monitoring tools.

These findings have several implications. For policymakers and healthcare administrators, they suggest the need for clearer sustainability requirements within public procurement (for example, through increasing use of Environmental Product Declarations), and the adoption of standards and protocols, such as ISO 50001 or Green Building Rating Systems. These findings also suggest the possibility of having dedicated funding mechanisms to address the implementation gap. From a research perspective, the study points to the value of developing more context-sensitive sustainability assessment tools for healthcare infrastructure, capable of supporting informed decision-making and guiding implementation in resource-constrained settings.

This exploratory study also has limitations. The empirical phase focused on a limited sample within a single regional context, which may not capture the full diversity of healthcare infrastructure or sustainability efforts across different geographies. Furthermore, data collection relied on available documents and self-reported information, which may be affected by variability in reporting quality and transparency, also in relation to the expertise of the respondent. Future research should expand the analysis to a broader national or international scale, explore longitudinal changes in hospital sustainability performance, and investigate the impact of specific interventions through case-based or quantitative approaches. Ultimately, advancing environmental sustainability in hospital infrastructure requires both technical innovation and institutional commitment, translating frameworks into practice and ambition into measurable outcomes.

## Figures and Tables

**Table 1:** Structure of the survey.

Environmental Criteria	Themes Addressed	Number of Questions
Energy Efficiency	Thermal and electrical energy demand, use, and production of energy from renewable sources, distribution systems, and energy management/monitoring.	9
Resource and waste management	Waste production and recycling, water consumption and reuse, water consumption management/monitoring systems, presence of green cover.	7
Transport and mobility	Presence of parking lots for light mobility and electric vehicle charging.	2
Materials and constructions	Presence of strategies/policies on recycled materials.	2
Environmental compliance	Environmental certifications and strategies ESGs for the achievement of SDGs	2
GHG Emissions	CO_2_ emissions	1
Strategies adopted in the design	Strategies adopted for climate change resilience and mitigation	3
Site Sustainability	Green areas and site integration	2

**Table 2:** Environmental sustainability dimensions analyzed in the literature.

Environmental Sustainability Dimensions	Key Themes Identified	Supporting References
1. Energy Efficiency	Energy Intensity (EUI), renewable energy, energy monitoring.	[21,22,23,24,25,26,27,28,29,30,31,32,33]
2. Resource consumption and waste management	Waste separation, hazardous waste management, and stormwater and wastewater reuse.	[21,24,25,28,34,35,36,37]
3. Transport and Mobility	Sustainable mobility policies, EV charging points, and reduction in private vehicle use.	[25,34,35,36,37]
4. Materials	Use of low-impact materials, and free of harmful materials.	[22,25,26,38]
5. Environmental Compliance	Adoption of environmental certifications and standards	[23,24,27,38]
6. GHG Emissions	CO_2_ reduction strategies; management of anesthetic gases.	[21,22,27,28,31,35,38,39]
7. Site Sustainability	Green and permeable areas	[26,40,41]
8. Design Strategies	Passive design, orientation, shading, green roof, and ventilating façades.	[22,26,28,30,31,32,40]

## Data Availability

The original contributions presented in this study are included in the article. Further inquiries can be directed to the corresponding author.

## Abbreviations

The following abbreviations are used in this manuscript:

CO_2_	Carbon Dioxide
SDGs	Sustainable Development Goals
LEED	Leadership in Energy and Environmental Design
BREEAM	Building Research Establishment Environmental Assessment Method
ISO	International Organization for Standardization
EMAS	Eco-Management and Audit Scheme
BEI	Building Energy Intensity
EUI	Energy Use Intensity
VOC	Volatile Organic Compounds
ESG	Environmental, Social, and Governance
POE	Post Occupancy Evaluation
DNSH	Do No Significant Harm
PNRR	Piano Nazionale di Ripresa e Resilienza (Italian Recovery Plan)
BEMS	Building Energy Management System
EPD	Environmental Product Declaration
PRISMA	Preferred Reporting Items for Systematic Reviews and Meta-Analyses
DEA	Dipartimento di Emergenza e Urgenza (Emergency Department)

## Appendix A. List of the Hospital Facilities Assessed

ASST Spedali Civili di Brescia
ASST Garda—Ospedale di Manebrio
ASST Garda—Ospedale di Leno
ASST Garda—Ospedale di Desenzano
ASST Garda—Ospedale di Gavardo
ASST Papa Giovanni XXIII
ASST Papa Giovanni XXIII—Ospedale San Giovanni Bianco
ASST Cremona—Ospedale di Cremona
ASST Cremona—Ospedale Oglio Po
ASST Sette Laghi—Ospedale Filippo del Ponte
ASST Sette Laghi—Ospedale Carlo Ondoli
ASST Sette Laghi—Ospedale Causa Pia Luvini
ASST Sette Laghi—Ospedale Calvarini
ASST Sette Laghi—Ospedale Luini Confalonieri
Istituto Auxiologico San Luca
Istituto Auxiologico Ospedale Capitanio
Istituto Auxiliologico Ospedale Mosè Bianchi

## Appendix B. Survey Structure

1. Key Information	1.1 Hospital facility:
	1.2 Name of contact person(s):
	1.3 Contact person(s):
	1.4 Gross floor area (total sqm):
	1.5 Number of beds:
2. Energy efficiency	2.1 Value of thermal energy requirement (if available in kWh/m^2^/year):
	2.2 Value of electricity requirement (if available in kWh/m^2^/year):
	2.3 Reference year of the above data:
	2.4 Does the facility use energy from renewable sources (not necessarily on-site)? If so, what is the percentage of total needs?
	2.5 Is energy produced from on-site renewable sources at the facility? If so, what is the percentage of total demand?
	2.6 Are there efficient energy distribution and management systems? Which ones?
	2.7 Is the Building Energy Management System (BEMS) used in the facility to constantly monitor energy consumption?
	2.8 If other monitoring systems are used, what are they?
	2.9 Does the facility feature facade and roofing systems / passive systems / natural ventilation systems that are useful for achieving energy efficiency?
3. Resource and waste management	3.1 How much waste is produced (Kg) per day or per year?
	3.2 Approximately what percentage of waste is recycled out of the total waste produced?
	3.3 How much water do you consume (litres or cubic metres) per day or per year?
	3.4 Is there a software or platform for monitoring and rational management of water consumption? If so, which one?
	3.5 Is there a system for purifying and reusing sanitary water consumed for secondary purposes?
	3.6 Is there a system for storing and reusing rainwater?
	3.7 Does the facility have a portion of green roof? If so, what is its percentage compared to the entire footprint of the building?
4. Transport and mobility	4.1 Is there a sustainable mobility sharing service?
	4.2 Are there bicycle and light mobility parking spaces? If so, how many?
	4.3 Are there parking spaces for charging electric vehicles? If so, how many?
5. Materials and constructions	5.1 Does the facility have internal strategies or policies regarding the percentage of recycled materials, in addition to those required by law?
	5.2 Is there a request for environmental product declaration (EPD) in the facility for all building materials and furnishings supplied/installed in the most recent interventions?
6. Environmental compliance	6.1 Does the facility have an environmental certification? If so, which one? (LEED Silver, Gold, BREEAM, etc.):
	6.2 Are there clear and adequate ESG responses to achieve the Sustainable Development Goals (SDGs) in the institution’s policies?
7. Emissions	7.1 Equivalent CO_2_ emission value expressed in Kg? If available, which year does it refer to?
8. Design strategies	8.1 Are there any climate resilience solutions, plans or policies in place? If so, which ones?
9. Site Sustainability	9.1 Please indicate the percentage of green areas relative to the hospital’s total site area
	9.2 In what percentage does the hospital site include permeable paved areas?
